# Differences in Exercise Capacity and Responses to Training in 24 Inbred Mouse Strains

**DOI:** 10.3389/fphys.2017.00974

**Published:** 2017-11-30

**Authors:** Joshua J. Avila, Seung Kyum Kim, Michael P. Massett

**Affiliations:** Department of Health and Kinesiology, Texas A&M University, College Station, TX, United States

**Keywords:** exercise training, treadmill running, heritability, inbred strains, muscle, heart

## Abstract

Changes in cardiorespiratory fitness in response to a standardized exercise training protocol differ substantially between individuals. Results from cross-sectional, twin, and family studies indicate genetics contribute to individual differences in both baseline exercise capacity and the response to training. Exercise capacity and responses to training also vary between inbred strains of mice. However, such studies have utilized a limited number of inbred strains. Therefore, the aim of this study was to characterize exercise-training responses in a larger number of genetically diverse strains of inbred mice and estimate the contribution of genetic background to exercise training responses. Eight-week old male mice from 24 inbred strains (*n* = 4–10/strain) performed a graded exercise test before and after 4 weeks of exercise training. Before training, exercise capacity was significantly different between strains when expressed as time (range = 21–42 min) and work performed (range = 0.42–3.89 kg·m). The responses to training also were significantly different between strains, ranging from a decrease of 2.2 min in NON/ShiLtJ mice to an increase of 8.7 min in SWR/J mice. Changes in work also varied considerably between the lowest (−0.24 kg·m in NON/ShiLtJ) and highest (+2.30 kg·m in FVB/NJ) performing strains. Heart and skeletal muscle masses also varied significantly between strains. Two broad sense heritability estimates were calculated for each measure of exercise capacity and for responses to training. For change in run time, the intraclass correlation between mice within the same inbred strain (*r*_I_) was 0.58 and the coefficient of genetic determination (*g*^2^) was 0.41. Heritability estimates were similar for the change in work: *r*_I_ = 0.54 and *g*^2^ = 0.37. In conclusion, these results indicate genetic background significantly influences responses to exercise training.

## Introduction

Cardiorespiratory fitness (i.e., endurance exercise capacity determined by a graded treadmill test) is a predictor of cardiovascular disease and all-cause mortality in men and women (Blair et al., 1989; Myers et al., 2002; Gulati et al., 2003; Kodama et al., 2009). Improvements in cardiorespiratory fitness are associated with significantly reduced risk of all-cause mortality (Blair et al., 1995; Erikssen et al., 1998; Kokkinos et al., 2010; Brawner et al., 2017). However, changes in cardiorespiratory fitness in response to a standardized exercise training protocol are variable (Lortie et al., 1984; Kohrt et al., 1991; Bouchard et al., 1999; Bouchard and Rankinen, 2001). The reported percentage of individuals showing minimal or no improvements in cardiorespiratory fitness in response to a standardized training program ranges from 7–45% (Sisson et al., 2009; Bouchard et al., 2011; Scharhag-Rosenberger et al., 2012). Bamman et al. used cluster analysis to identify non-, modest, and extreme responders for resistance training-induced skeletal muscle hypertrophy, demonstrating that skeletal muscle adaptations to resistance training also vary between individuals (Bamman et al., 2007; Thalacker-Mercer et al., 2013). Although individual variation in training responses is being widely investigated, the mechanisms underlying this individual variation are not fully understood.

Accumulating evidence suggests that a genetic component contributes to the variability in exercise capacity and the responses to exercise training. Based on comparisons between families, the between family variance is 2–3 times greater than within family variance for maximal oxygen consumption (VO_2max_) and sub-maximal power output (Bouchard et al., 1999). Less is known about the genetic basis for training responses. The HERITAGE Family Study reported a heritability of 47% for the change in VO_2max_ with training (Bouchard et al., 1999). Furthermore, monozygotic twins show a high resemblance in training responses expressed as changes in VO_2max_ (Prud'homme et al., 1984; Hamel et al., 1986; Zadro et al., 2017). Pooled estimates of monozygotic twin pair correlations range from 0.38 to 0.74 for changes in VO_2max_, supporting a genetic contribution to variation in exercise training responses. Collectively, those data indicate there is heterogeneity in both intrinsic exercise capacity and exercise training responses, which is dependent, in part, on genotype.

Despite evidence of a genetic component to exercise capacity and responses to training, the need for large sample sizes and variation in training paradigms have limited replication of pertinent results in humans (Hagberg et al., 2011). Thus, alternative approaches are needed to investigate the genetic basis for variation in responses to exercise training. One approach is to use genetically defined model organisms such as inbred or selectively bred strains of rodents to identify potential modifiers of the response to exercise training (Troxell et al., 2003; Massett and Berk, 2005; Rezende et al., 2005, 2006; Massett et al., 2009, 2015; Meek et al., 2009; Kilikevicius et al., 2013; Koch et al., 2013). Responses to training are variable between inbred strains of rats as well as rats selectively bred for high and low responses to training (Koch et al., 2013). Similarly, endurance exercise capacity and maximal oxygen consumption differ between mice selectively bred for high wheel running activity and control lines (Rezende et al., 2005, 2006; Meek et al., 2009). A small number of inbred and hybrid mouse strains have been utilized to investigate the genetic basis for changes in exercise capacity following treadmill running or swim training (Massett and Berk, 2005; Massett et al., 2009, 2015; Kilikevicius et al., 2013). In those studies, training responses varied significantly between mouse strains. Broad sense heritability estimates ranged from 0.38 to 0.56 for changes in exercise capacity assessed by treadmill running (Massett and Berk, 2005), suggesting that variation in adaptation responses to exercise in mice is significantly influenced by genetic background. Those studies utilized a diverse, but smaller number of inbred strains, which potentially limited the range of phenotypic differences. Surveying a larger number of strains with wider genetic diversity could identify greater heterogeneity at the phenotype level. Large differences in responses between genetically diverse strains can serve as a starting point for more detailed genetic (i.e., linkage analysis or genome-wide association studies) and physiological studies of the mechanisms underlying variation in responses to exercise training. Therefore the aim of the current study was to characterize exercise-training responses in 24 strains of inbred mice. The contribution of genetic background to the responses to exercise training also was estimated on this genetically diverse population of mice.

## Materials and methods

### Animals

This study was carried out in accordance with the recommendations of the National Institutes of Health guidelines for the care and use of laboratory animals. The protocol was approved by the Institutional Animal Care and Use Committee at Texas A&M University. Six to seven week old male mice from 24 inbred strains (*n* ≈ 8–18/strain) were purchased from Jackson Laboratories and allowed to acclimatize to their housing for at least 1 week upon arrival. The following strains were used: 129S1/SvImJ, 129X1/SvJ, A/J, AKR/J, BALB/cByJ, C3H/HeJ, C57BL/6J, C57BR/cdJ, CBA/J, CE/J, DBA/2J, FVB/NJ, I/LnJ, LG/J, LP/J, MA/MyJ, NOD/ShiLtJ, NON/ShiLtJ, NZW/LacJ, PL/J, PWD/PhJ, SJL/J, SM/J, SWR/J. These strains were chosen based on their phylogenetically distinct background (Petkov et al., 2004) and the recommendations of the Mouse Phenome Database (Grubb et al., 2014) to include a wide range of genetic diversity. Eight week old, young adult mice were chosen for this study because the rapid rate of postnatal growth plateaus around this age (Gall and Kyle, 1968; Eisen, 1976). Wheel running activity also peaks near this age in mice (Swallow et al., 1998) and declines after 10 weeks of age in multiple strains of inbred mice (Turner et al., 2005). Furthermore, multiple studies have utilized mice within this age range for assessing exercise capacity (Lightfoot et al., 2001; Kemi et al., 2002; Lerman et al., 2002), responses to training (Kemi et al., 2002), or locomotor activity (Kelly et al., 2010). Therefore, selecting mice within this age range coincides with their peak voluntary activity levels and permits comparisons with previous studies, including our own. All mice were group housed in standard caging and allowed food (Standardized Laboratory Rodent Diet) and water *ad libitum* and maintained at an ambient temperature of 22–24°C on a 12 h light:dark schedule.

### Exercise performance test

At 8 weeks of age, all mice were familiarized to running on a motorized rodent treadmill (Columbus Instruments, Columbus, OH) for 2 days as described previously (Desai et al., 1997; Massett et al., 2009, 2015). Each session was approximately 10 min in duration and mice ran at 9 and 10 m per minute (m/min) up a 10° incline. After familiarization, mice performed two graded exercise performance tests separated by 48 h. Tests started at 9 m/min for 9 min then increased from 10 m/min by 2.5 m/min every 3 min. The starting incline was 0° and was raised by 5° every 9 min, with a maximal incline of 15°. Exercise continued until exhaustion, defined as spending greater than 15 consecutive seconds on the electric grid at the rear of the treadmill. At this point, running time (in min) was recorded and each mouse was removed from the treadmill and returned to its home cage. Exercise capacity was expressed in time (min) and work (kg·m). Work performed (kg·m) was calculated as the product of body weight (kg) and vertical distance (meters), where vertical distance = (distance run)(sin θ), where θ is equal to the angle of the treadmill from 0° to 15° (Barbato et al., 1998; Massett et al., 2009, 2015). A second pair of exercise performance tests was completed after the training period and changes in exercise capacity were calculated. For each mouse, the average of the two trials at each time point were used to calculate pre (or baseline) and post intervention exercise capacity, respectively. For one strain, SWR, three baseline exercise tests were performed because the difference between tests 1 and 2 was approximately 7 min. The average of three trials was used for SWR strain mean calculations. Overall mean differences between test 1 and test 2 for both groups at both time points were less than 1 min (Pre: EX = 0.2 ± 2.0 min, SED = 0.7 ± 2.9 min; Post: EX = −0.7 ± 2.4 min, SED = 0.3 ± 2.3 min). Therefore, average values from the repeated trials were used in subsequent analyses.

### Exercise training

The exercise training program was designed to match those previously reported by our laboratory (Massett and Berk, 2005; Massett et al., 2009, 2015) and to match the exercise recommendations for optimal fitness in humans (Haskell et al., 2007; Garber et al., 2011). This protocol and similar protocols have been shown to produce the expected cardiovascular and skeletal muscle adaptations (De Angelis et al., 2004; Massett and Berk, 2005; Al-Jarrah et al., 2007; Massett et al., 2009; Savage and McPherron, 2010; Meier et al., 2013). Before the initial exercise tests, mice were randomly assigned to exercise training (EX, *n* = 4–10/strain) or sedentary control (SED, *n* = 4–8/strain) groups. EX mice performed 4 weeks of exercise training on a six lane rodent treadmill (Columbus Instruments), running 5 days/week for 60 min/day. The target workload for the training protocol, 65% of the maximal work-load attained during the exercise performance test, was based on the strain mean for the pre-training exercise performance test (Massett et al., 2009, 2015). The average relative workload for all mice was 64.8 ± 3.7% (95% CI: 64.1–65.3%) of the maximal workload. A moderate intensity of 65% of maximal workload was chosen to ensure that all strains would be able to complete the protocol over 4 weeks. The relative workload (% of maximum) was chosen to account for differences in intrinsic exercise capacity between strains. During the first 2 weeks of exercise training the speed and duration were gradually increased until the desired training workload could be sustained. SED mice performed pre and post exercise tests and were handled weekly but not made to run. All SED mice repeated the familiarization protocol at least 2 days prior to the post exercise tests.

### Body and tissue mass

Body mass was recorded in grams before and after the training period. At least 24 h after the last exercise test all mice were weighed and anesthetized with an intraperitoneal injection with Ketamine (80 mg/kg) – Xylazine (5 mg/kg) cocktail. Heart, gastrocnemius, plantaris, and the soleus muscle were harvested, washed in ice-cold (4°C) saline and weighed (wet weight in mg).

### Heritability

Estimates of broad sense heritability were calculated based on intraclass correlation (*r*_*I*_), which is an estimate of the proportion of the total phenotype variation that is accounted for by differences between strains, and the coefficient of genetic determination (*g*^2^), which accounts for the doubling of the additive genetic variance that occurs with inbreeding (Festing, 1979; Falconer, 1989; Lightfoot et al., 2001). The following equations were used to calculate *r*_*I*_ and *g*^2^: *r*_*I*_ = (MS_B_ – MS_W_)/[MS_B_ + (*n* – 1)MS_W_] and *g*^2^ = (MS_B_ – MS_W_)/[MS_B_ + (2*n* – 1)MS_W_], where MS_B_ and MS_W_ are the between- and within- mean square, respectively, and *n* is the number of animals per strain.

### Statistical analysis

All phenotype data were log transformed before analyses. After inspection of residuals, one mouse with a studentized residual value of −4.95 for change in time was identified as an outlier (C3H/HeJ, SED group) and eliminated from further analyses. Because of missing anthropometric data for the EX group from the BALB/cByJ strain, data from both the EX and SED groups were excluded from analyses of anthropometric phenotypes. Phenotype data were analyzed using two-way ANOVA with strain and group (EX and SED) as independent variables. For any phenotype with a significant strain × group interaction, strain comparisons within the EX and SED groups were made using a one-way ANOVA followed by a Tukey HSD test. Analysis of covariance was conducted to determine a difference between strains and groups (EX and SED) on changes in exercise time and work controlling for baseline exercise capacity. Pairwise comparisons were made using a Tukey HSD test using a Bonferroni corrected *P*-value of 0.001. For phenotypic correlations, all possible pairs between individual exercise and anthropometric variables were analyzed by Pearson correlation. For genetic correlations (Crabbe et al., 1990), all possible pairs between strain means of exercise and anthropometric variables were analyzed by Pearson correlation. All data are presented as mean ± SD. Statistical significance was set at P < 0.05, unless noted otherwise. All statistical analyses were performed using JMP Pro 13.1.0 (SAS Institute Inc., Cary, NC) or Prism 5.0 (GraphPad Software, Inc., La Jolla, CA).

To assess repeatability of exercise performance in mice over the 4 week period, typical error (TE) was calculated from pre and post tests in the SED group. TE was calculated for change in time (in min) and change in work (in kg·m) using the following equation: TE = SD_diff_ / $\sqrt{2}$ where SD_diff_ is the standard deviation of the difference scores between post and pre tests (Hopkins, 2000). TE = 2.30 min for the change in time and TE = 0.45 kg·m for the change in work. A strain exhibiting a response greater than TE was considered as having a potential positive or negative response to training beyond that due to technical error and/or biological variation. Strains exhibiting responses 2 times the TE were considered as have a real physiological response to training (Hopkins, 2000). To assess the magnitude of the training response, effect size was calculated using mean differences between EX and SED groups for change in time and change in work with thresholds set as *d* = 0.2 for small, *d* = 0.5 for medium, and *d* = 0.8 for large (Cohen, 1988).

## Results

### Exercise capacity and changes with training

**Figure 1:**
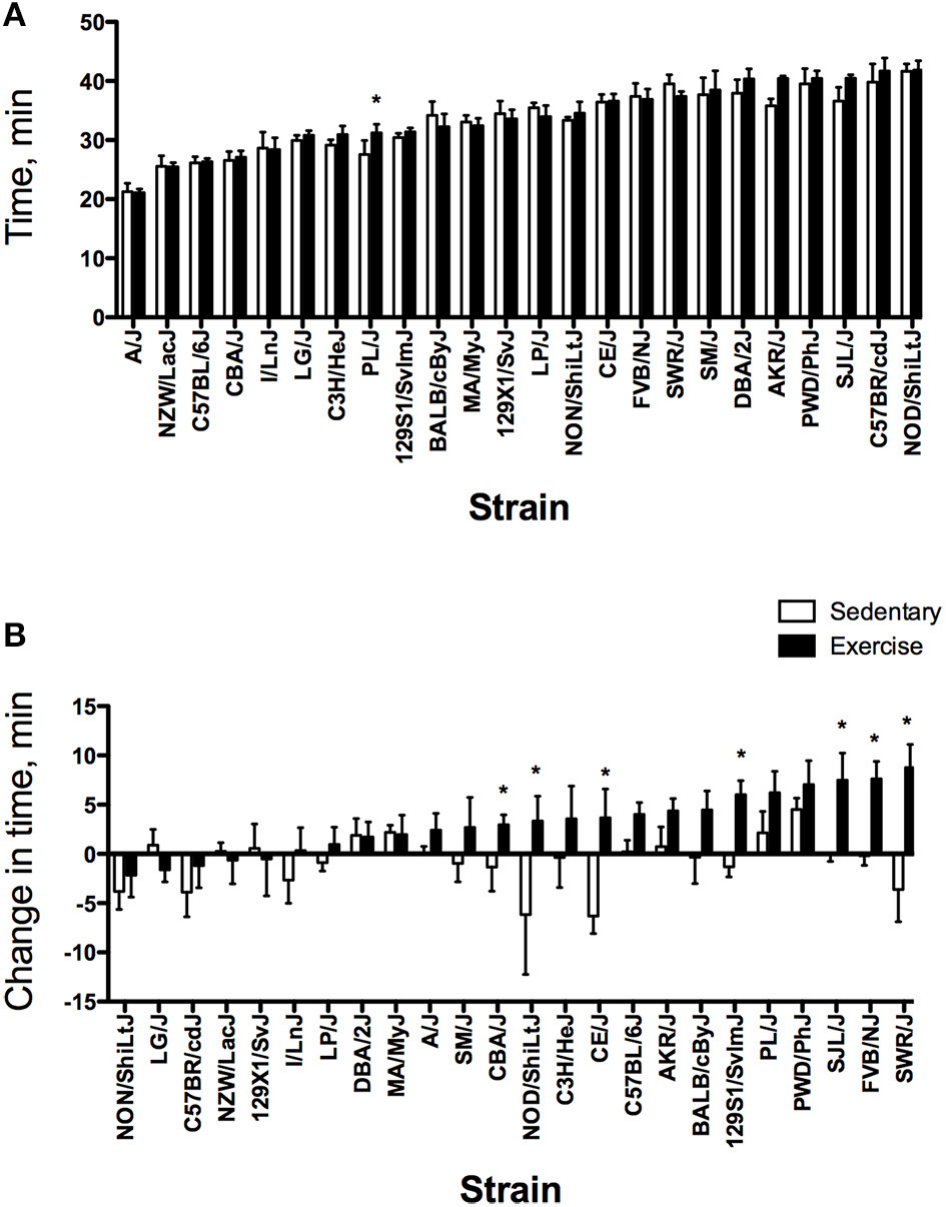
Strain distribution pattern for pre-training (baseline) exercise time **(A)** and change in time **(B)** in minutes for sedentary (SED) and exercise-trained (EX) mice from 24 inbred strains. For each phenotype, strains are sorted from lowest to highest based on the exercise group. Values are expressed as mean ± SD. ^*^P < 0.05 compared with SED (from ANOVA and Tukey HSD). 129S1/SvImJ (*n* = 6 for EX, *n* = 6 for SED), 129X1/SvJ (6, 6), A/J (6, 5), AKR/J (5, 5), BALB/cByJ (6, 6), C3H/HeJ (6, 5), C57BL/6J (6, 6), C57BR/cdJ (6, 6), CBA/J (6, 6), CE/J (6, 6), DBA/2J (6, 6), FVB/NJ (10, 8), I/LnJ (6, 5), LG/J (4, 4), LP/J (6, 6), MA/MyJ (6, 6), NOD/ShiLtJ (6, 6), NON/ShiLtJ (6, 6), NZW/LacJ (6, 6), PL/J (6, 6), PWD/PhJ (6, 5), SJL/J (6, 6), SM/J (6, 6), SWR/J (5, 5).

**Figure 2:**
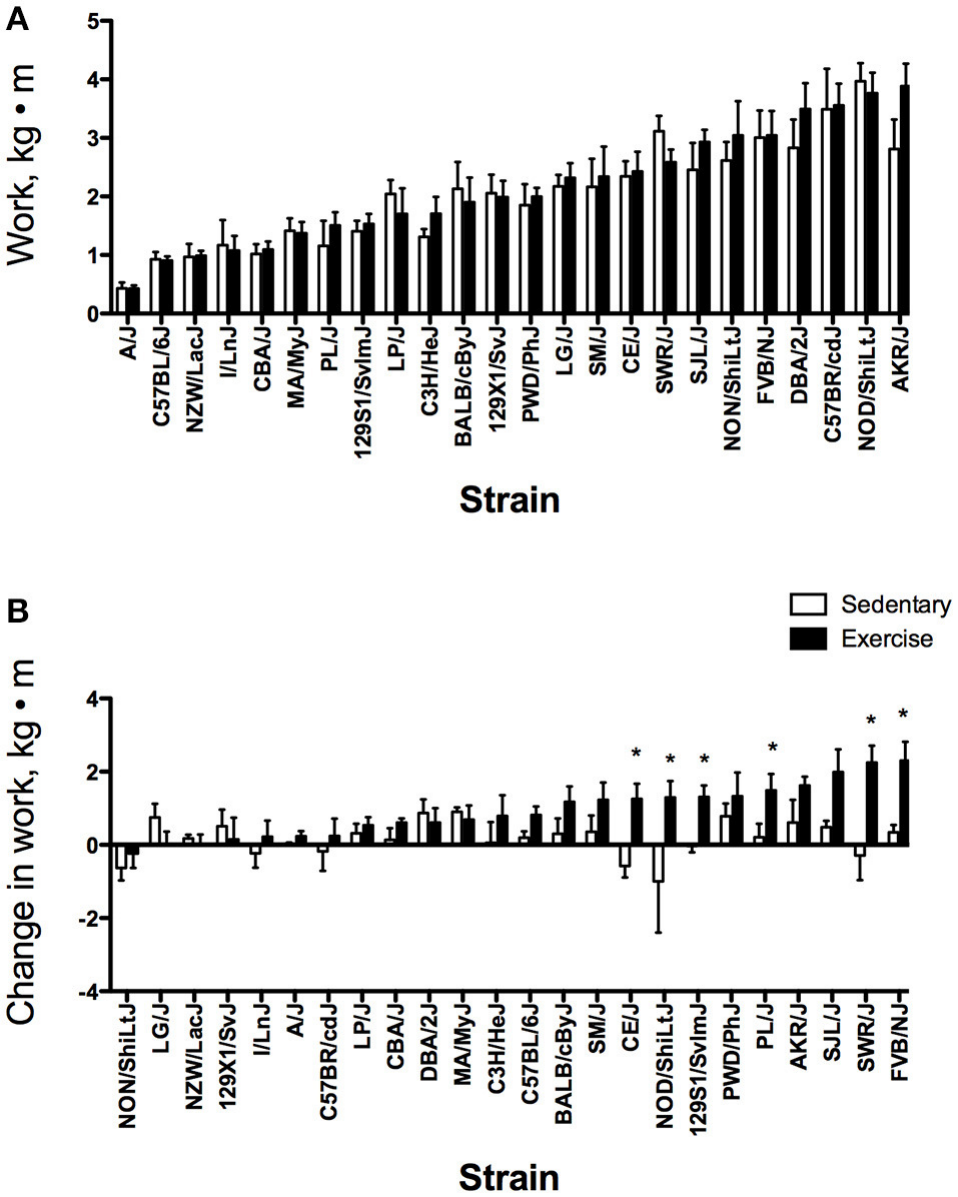
Strain distribution pattern for pre-training (baseline) work **(A)** and change in work **(B)** in kg·m for sedentary (SED) and exercise-trained (EX) mice from 24 inbred strains. For each phenotype, strains are sorted from lowest to highest based on the exercise group. Values are expressed as mean ± SD. *n* = 4–8/strain for SED and *n* = 4–10/strain for EX. ^*^P < 0.05 compared with SED (from ANOVA and Tukey HSD).

For all exercise phenotypes, main effects (strain and group) and the interaction (strain × group) were significant; therefore, strain comparisons were made within each group (EX and SED). Results for all exercise phenotypes will be described by group before comparing EX and SED groups. Strain distributions for pre-training exercise capacity expressed as time and work are shown in Figures 1A, 2A, respectively. For EX mice, pre-training exercise capacity ranged from 21.1 ± 0.6 min to 41.8 ± 1.6 min (*P* < 0.0001) between the lowest and highest performing strains. For pre-training work, maximal work in the lowest performing strain was only 11% of that in the highest performing strain (0.42 ± 0.06 kg·m vs. 3.89 ± 0.38 kg·m) (Figure 2A, *P* < 0.0001). When examining the strain distribution pattern in EX mice for pre-training time and work, AKR/J, DBA/2J, C57BR/cdJ, and NOD/ShiLtJ strains were in the upper quartile for both phenotypes, whereas A/J, CBA/J, C57BL/6J, I/LnJ, and NZW/LacJ strains were in the lowest quartile for both phenotypes. After 4 weeks of exercise training, the difference between the highest (48.0 ± 2.9 min) and lowest performing strains (23.5 ± 2.0 min) was approximately 25 min (*P* < 0.0001) when exercise capacity is expressed as time (Data not shown). Similar to the pattern seen in pre-training work, there was a significant difference between strains for post-training work with values ranging from 0.68 ± 0.16 kg·m to 5.51 ± 0.40 kg·m. When examining differences in training responses between strains, change in time in EX mice ranged from −2.2 ± 2.2 min to +8.7 ± 2.3 min (*P* < 0.0001) (Figure 1B). The change in work ranged from a low of −0.24 ± 0.39 kg·m to a high of 2.30 ± 0.51 kg·m (Figure 2B, *P* < 0.0001). Fifteen strains had a mean increase in time that exceeded TE (2.30 min), while six of those strains (129S1, FVB/NJ, PL/J, PWD/PhJ, SJL/J, and SWR/J) had an increase in time greater than two times TE. Similarly for work, mean increases in work in 17 strains exceeded TE (0.45 kg·m). Mean increases in work for 11 of those strains (129S1/SvlmJ, AKR/J, BALB/cByJ, CE/J, FVB/NJ, NOD/ShiLtJ, PWD/PhJ, PL/J, SJL/J, SM/J, and SWR/J) were greater than two times TE. Changes greater than twice the TE have a high probability of being a true physiological adaptation beyond that associated with biological and technical variability (Hopkins, 2000). The significant difference between inbred strains for changes in exercise capacity implies that the response to training is determined, in part, by genetics.

As with EX mice, there were significant differences between strains for time (*P* < 0.0001) and work (*P* < 0.0001) for SED mice at the start of the 4-week period. The differences between the lowest and highest performing strains for time and work were 20 min and 3.54 kg·m, respectively. These ranges were similar to those observed in EX mice (Figures 1A, 2A). After the 4-week period, post time and work in SED mice also varied between strains with ranges of 21.3 ± 1.4 min to 44.0 ± 2.5 min for time and 0.46 ± 0.10 kg·m to 3.70 ± 0.41 kg·m for work (Data not shown). The change in responses over time between strains for SED mice ranged from −6.3 ± 1.7 min to +4.5 ± 1.2 min for exercise time. The change in work between strains for SED mice also differed significantly between strains (−0.99 ± 1.40 kg·m to 0.90 ± 0.12 kg·m). Mean changes in exercise time exceeded TE in seven strains (6 decrease, 1 increase), with two of these strains having reductions in time greater than two times TE (CE/J and NOD/ShiLtJ). The mean decrease in work in NOD/ShiLtJ strain also exceeded two times TE. A total of 10 strains had mean changes in work greater than TE (3 decrease, 7 increase).

In comparing SED and EX groups, the main effect for group was significant for exercise time (*P* = 0.0006) and work (*P* = 0.0035) for baseline endurance exercise capacity. However, for the majority of strains, there was no significant difference between SED and EX groups (Figures 1A, 2A). In contrast, most strains differed between SED and EX groups for endurance exercise capacity measured after the intervention period (Data not shown). Significant differences for the change in time between SED and EX groups were found in seven strains (Figure 1B). SED and EX groups from six strains differed significantly for changes in exercise capacity expressed as work (Figure 2B). Overall, the EX group increased exercise time with training (3.21 ± 3.69 min), while the SED group had a mean decrease in time (−0.79 ± 3.25 min). Mean changes in work for EX and SED mice after 4 weeks were 0.97 ± 0.82 kg·m and 0.16 ± 0.64 kg·m, respectively. The standardized mean effect for change in time was *d* = 0.65 and *d* = 0.52 for the change in work, approximately equal to a medium effect for the response to training.

The large range of values for pre-training exercise capacity raised the question that mice with low initial values would have the greatest responses to training, i.e., “initial value” principle. Therefore, analysis of covariance was performed to assess the influence of baseline exercise capacity on the change in exercise capacity. For change in exercise time, there were significant effects of strain (*F* = 15.3, *P* < 0.0001, eta^2^ = 33.5), group (*F* = 274.6, *P* < 0.0001, eta^2^ = 26.2), strain × group interaction (*F* = 8.8, *P* < 0.0001, eta^2^ = 19.4), and baseline exercise time (*F* = 72.6, *P* < 0.0001, eta^2^ = 6.9). Baseline exercise time had an overall negative effect on the change in time (ß = −0.63, SE = 0.07, *P* < 0.0001). Least squares means ± 99.9% CI for the change in exercise time is shown in Supplemental Table 1. Seven strains showed a significant increase in exercise capacity that was different from changes in time in SED controls (129S1, CE/J, FVB/NJ, NOD/ShiLtJ, PL/J, SJL/J, and SWR/J) (*P* < 0.001). A similar analysis for work found significant effects of strain (*F* = 12.3, *P* < 0.0001, eta^2^ = 30.6), group (*F* = 199.7, *P* < 0.0001, eta^2^ = 21.6), strain × group interaction (*F* = 8.3, *P* < 0.0001, eta^2^ = 20.6), and baseline work (*F* = 81.4, *P* < 0.0001, eta^2^ = 8.8). Baseline work also had a negative effect on change in work (ß = −0.60, SE = 0.07, *P* < 0.0001). Least squares means ± 99.9% CI for the change in exercise time is shown in Supplemental Table 2. After adjusting for baseline work, seven strains showed a significant increase in exercise capacity that was different from changes in work in SED controls (129S1, C3H/HeJ, CE/J, FVB/NJ, NOD/ShiLtJ, PL/J, and SWR/J) (*P* < 0.001).

### Anthropometric variables

**Figure 3:**
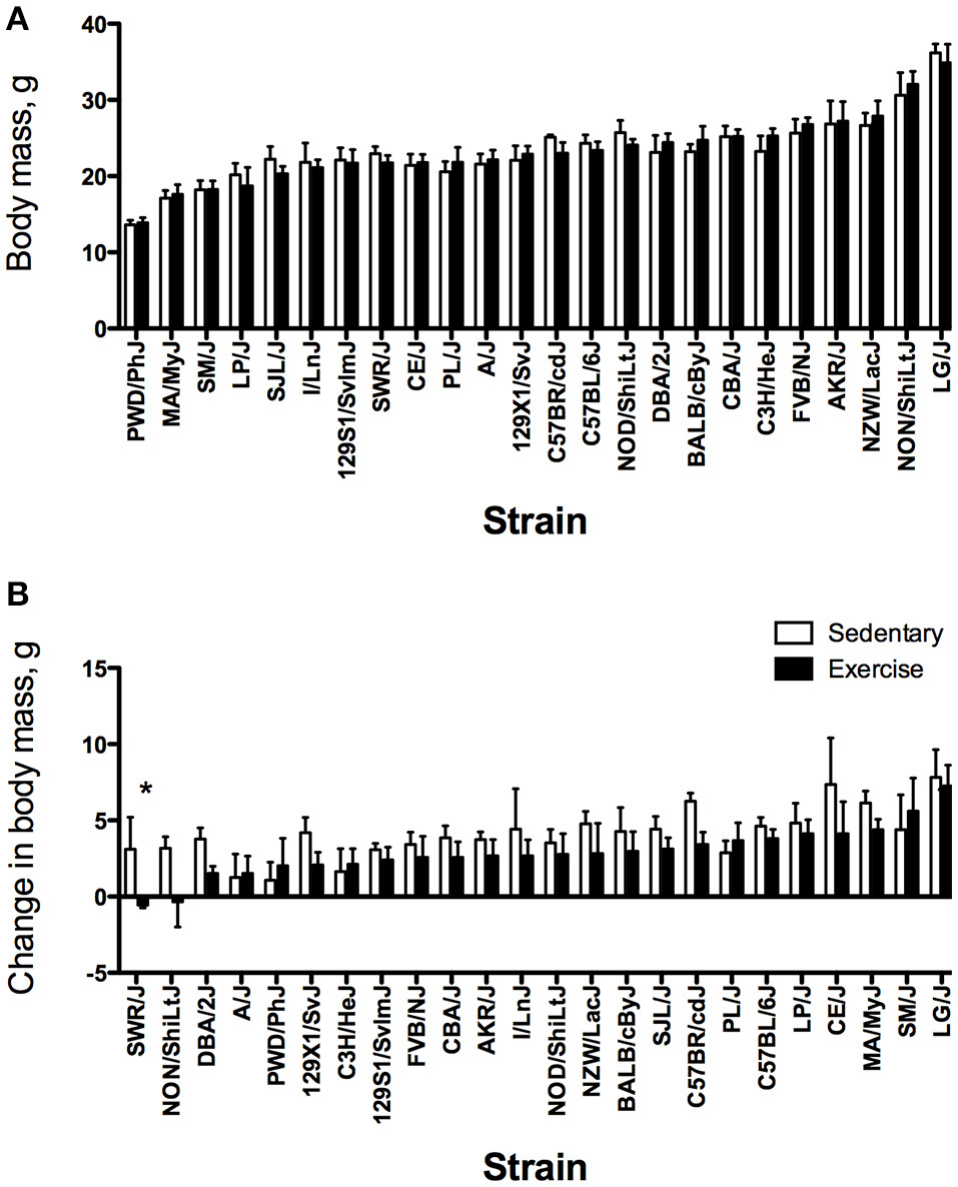
Strain distribution pattern for pre-training (baseline) body mass **(A)** and change in body mass **(B)** in grams for sedentary (SED) and exercise-trained (EX) mice from 24 inbred strains. For each phenotype, strains are sorted from lowest to highest based on the exercise group. Values are expressed as mean ± SD. *n* = 4–8/strain for SED and *n* = 4–10/strain for EX. ^*^P < 0.05 compared with SED (from ANOVA and Tukey HSD).

Body mass varied significantly between strains before and after the training period (Figures 3A,B) in both SED and EX groups. At the start of the study, body mass was approximately 2.5 times higher in the strain with the largest body mass compared with the smallest. After 4 weeks, the strain with the largest body mass was 2.5 to 3 times heavier than the strain with the lowest body mass. Body mass was not significantly different between groups before training (*P* = 0.77), but was different after training (*P* < 0.0001). Regardless of training group, LG/J mice had the highest body mass before and after training, whereas PWD/PhJ mice had the lowest. Changes in body mass over the 4-week training period also varied significantly between strains (*P* < 0.0001) (Figure 3B) with LG/J mice exhibiting the largest change in body mass irrespective of group. Overall, the increase in body mass was smaller in EX mice (2.8 ± 1.9 g) compared with SED mice (4.0 ± 2.1 g) (*P* < 0.0001), with only one strain (SWR/J) having a significant difference between EX and SED groups (*P* < 0.05) (Figure 3B).

**Figure 4:**
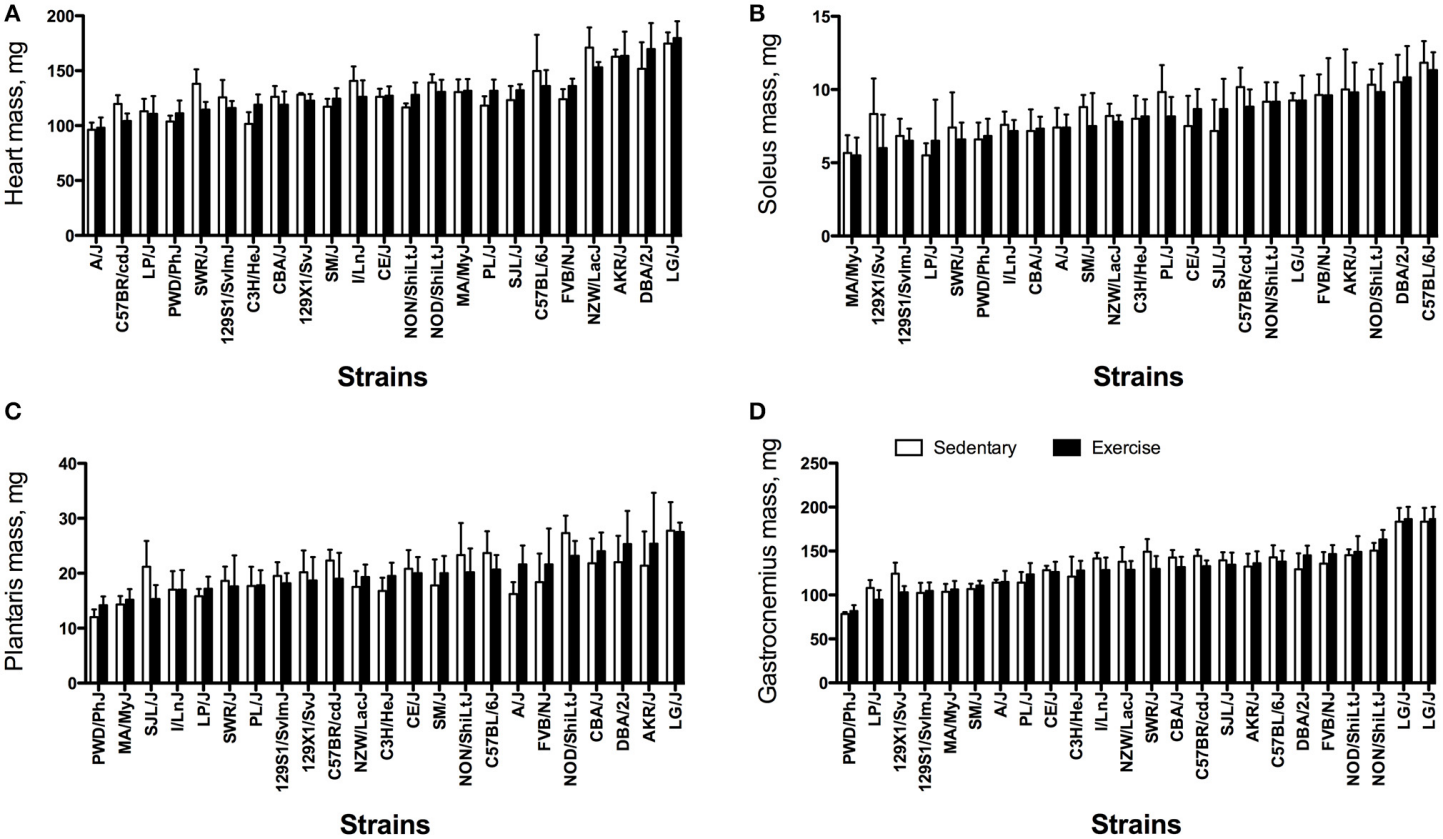
Heart and muscle masses from sedentary (SED) and exercise-trained (EX) mice from 23 inbred strains. **(A)** Heart mass, mg; **(B)** Soleus muscle mass, mg; **(C)** Plantaris muscle mass, mg; and **(D)** Gastrocnemius muscle mass, mg. Samples were obtained post-intervention. For each phenotype, strains are sorted from lowest to highest based on the exercise cohort. Values are expressed as mean ± SD. *n* = 4–8/strain for SED and *n* = 4–10/strain for EX.

**Figure 5:**
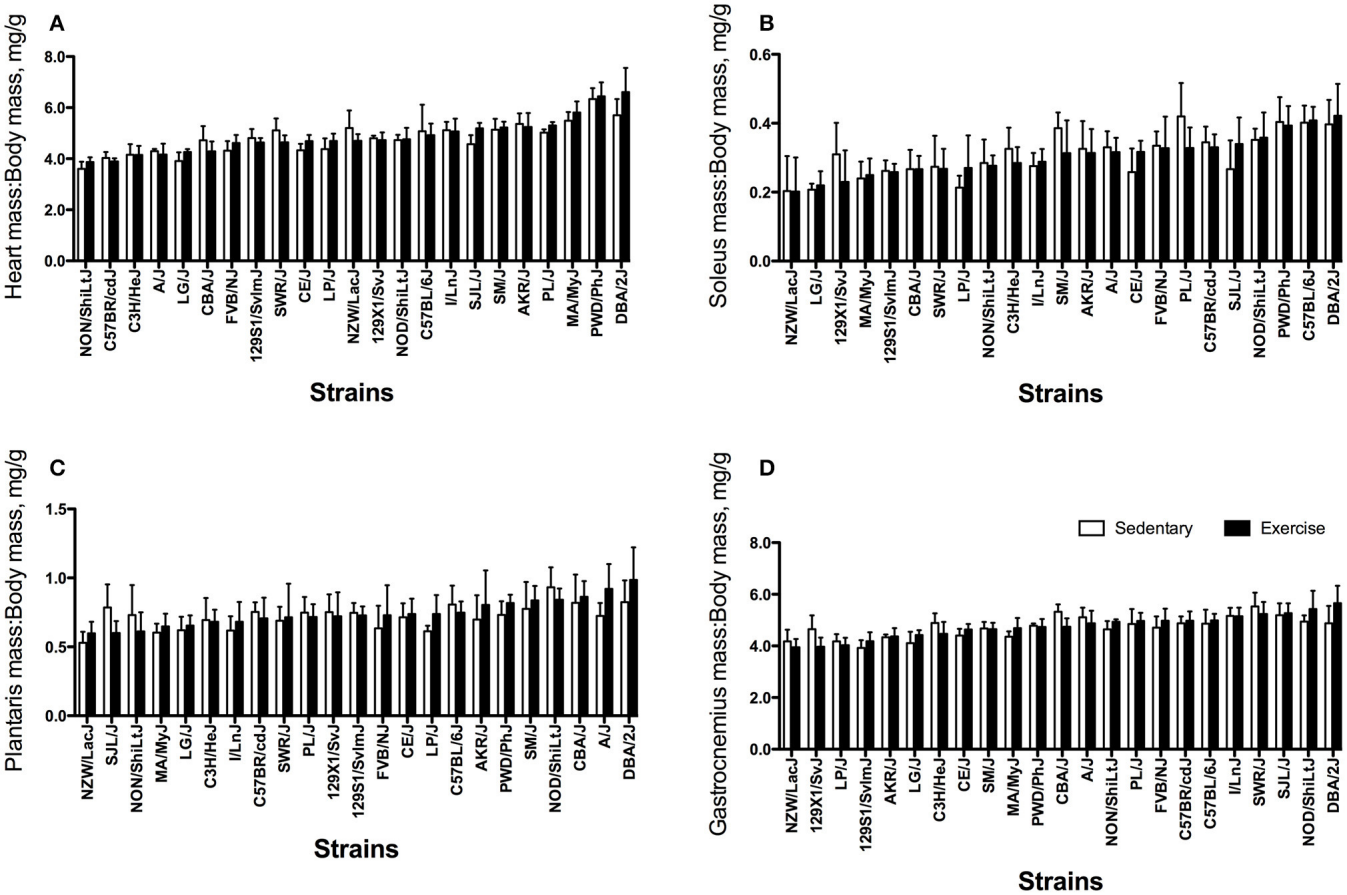
Heart and muscle masses relative to body mass from sedentary (SED) and exercise-trained (EX) mice from 23 inbred strains. **(A)** Heart mass-to-body mass ratio, mg/g; **(B)** Soleus muscle mass-to-body mass ratio, mg/g; **(C)** Plantaris muscle mass-to-body mass ratio, mg/g; and **(D)** Gastrocnemius muscle mass-to-body mass ratio, mg/g. Samples were obtained post-intervention. For each phenotype, strains are sorted from lowest to highest based on the exercise cohort. Values are expressed as mean ± SD. *n* = 4–8/strain for SED and *n* = 4–10/strain for EX.

There were significant strain effects for heart and muscle masses (Figure 4) as well as tissue mass corrected for body mass (Figure 5). On average, the heaviest tissue mass (heart or muscle) was double that of the lightest between strains in both the EX and SED groups. LG/J, NOD/ShiLtJ, and DBA/2J mice tended to have the largest tissue masses. PWD/PhJ, LP/J, and MA/MyJ mice had the smallest tissue masses irrespective of training group. For tissue mass-to-body mass ratios, DBA/2J mice had the highest ratios for most, whereas LG/J, MA/MyJ, and NZW/LacJ had the lowest ratios. Although the main effect for group (SED vs. EX) was not significant for any of the heart/muscle phenotypes, the strain by group interaction was significant for heart mass (*P* < 0.0001), plantaris mass (*P* = 0.049), gastrocnemius muscle mass (*P* = 0.009), heart mass-to-body mass ratio (*P* = 0.013), and gastrocnemius mass-to-body-mass ratio (*P* = 0.015).

### Heritability and correlations

Broad-sense heritability estimates were calculated for each measure of exercise capacity and anthropometric variables in the EX group mice (Table 1). The intraclass correlations (*r*_*I*_) for pre- and post-training exercise capacity were greater than 0.90, whereas the *r*_*I*_ for the response to training was 0.58 for change in time and 0.54 for change in work. The coefficients of genetic determination (*g*^2^) for these phenotypes were lower; approximately 0.80 for exercise capacity and 0.40 for responses to training. Broad sense heritability estimates for body mass and the change in body mass with training were comparable to those for the exercise phenotypes. Heritability estimates for tissue and muscle masses were somewhat lower and more wide-ranging than those for body mass or the exercise phenotypes (Table 1).

**Table 1:** Estimates of broad-sense heritability for exercise and anthropometric phenotypes for EX group mice.

**Phenotype**	***r*_*I*_**	***g*^2^**
Time, min		
Pre-training	0.94	0.88
Post-training	0.92	0.85
Change	0.58	0.41
Work, kg·m		
Pre-training	0.93	0.86
Post-training	0.90	0.82
Change	0.54	0.37
Body mass, g		
Pre-training	0.92	0.86
Post-training	0.87	0.77
Change	0.56	0.39
Heart mass, mg	0.70	0.54
Soleus mass, mg	0.38	0.23
Plantaris mass, mg	0.34	0.21
Gastrocnemius mass, mg	0.78	0.65
Heart mass:Body mass, mg/g	0.77	0.62
Soleus mass:Body mass, mg/g	0.31	0.18
Plantaris mass:Body mass, mg/g	0.20	0.11
Gastrocnemius mass:Body mass, mg/g	0.50	0.34

*r_I_, intraclass correlation coefficient; g^2^, coefficient of genetic determination. Estimates are based on data from 24 strains for exercise and body mass phenotypes and 23 strains for anthropometric phenotypes*.

Phenotypic and genetic correlations for EX mice are shown in Table 2. Overall, phenotypic and genetic correlations were similar. The response to training (i.e., change in time) was not correlated with pre-training time (*r* = 0.08), but significantly correlated with post-training time for both genetic (*r* = 0.48, *P* = 0.03) and phenotypic (*r* = 0.49, *P* < 0.0001) correlations. Exercise phenotypes were negatively correlated with body mass regardless of time point. The change in exercise time was significantly correlated with pre- (*r* = −0.24, *P* = 0.004) and post-training body mass (*r* = −0.29, *P* = 0.0007), but not the change in body mass (*r* = −0.04, *P* = 0.6). The genetic correlation between change in time and post-training body mass was significant (*r* = −0.46, *P* = 0.03). Exercise—tissue mass correlation coefficients were relatively small with no significant correlations between the change in time and tissue mass. Heart- and muscle mass-to-body mass ratios were more strongly correlated with exercise phenotypes. Significant genetic correlations were found between change in time and soleus-mass-to-body mass ratio and between post-training time and soleus-mass-to-body mass and gastrocnemius mass-to-body mass ratios. For phenotypic correlations, these two phenotypes were significantly correlated with all exercise phenotypes. Heart mass-to-body mass ratio was also significantly correlated with pre and post exercise time. Collectively, these results suggest that exercise capacity is inversely related to body mass and directly related to heart mass and muscle mass corrected for body mass.

**Table 2:** Genetic (above the diagonal) and phenotypic (below the diagonal) correlations for exercise and anthropomorphic phenotypes in EX group mice.

	**Exercise Time, min**			**Body Mass, g**			**Tissue Mass, mg**				**Tissue Mass:Body Mass ratio, mg/g**			
	**Pre**	**Post**	**Change**	**Pre**	**Post**	**Change**	**Heart**	**Soleus**	**Plantaris**	**Gastroc**.	**HM:BM**	**SM:BM**	**PM:BM**	**GM:BM**
Pre-training time		**0.92**	0.08	−0.20	−0.20	0.06	0.14	0.16	−0.12	0.02	0.35	0.37	0.13	0.40
Post-training time	**0.87**		**0.48**	−0.34	−0.36	0.02	0.03	0.14	−0.21	−0.09	0.41	**0.50**	0.22	**0.48**
Change in time	0.00	**0.49**		−0.39	−**0.46**	−0.08	−0.24	0.00	−0.27	−0.27	0.26	**0.43**	0.26	0.31
Pre-training body mass	−**0.18**	−**0.28**	−**0.24**		**0.94**	−0.40	**0.54**	**0.55**	**0.75**	**0.86**	−**0.68**	−**0.48**	−**0.52**	−0.39
Post-training body mass	−**0.17**	−**0.29**	−**0.29**	**0.91**		−0.07	**0.62**	**0.54**	**0.76**	**0.83**	−**0.55**	−**0.43**	−**0.42**	−0.33
Change in body mass	0.06	0.03	−0.04	−**0.43**	−0.02		0.08	−0.17	−0.13	−0.28	**0.53**	0.25	0.40	0.25
Heart mass	0.11	0.03	−0.14	**0.48**	**0.56**	0.06		**0.51**	**0.53**	**0.59**	0.24	−0.03	−0.16	−0.01
Soleus mass	0.12	0.11	0.01	**0.40**	**0.41**	−0.08	**0.39**		**0.61**	**0.74**	−0.21	**0.47**	−0.04	0.22
Plantaris mass	−0.09	−0.15	−0.15	**0.48**	**0.52**	−0.03	**0.39**	**0.43**		**0.68**	−**0.42**	−0.15	0.18	−0.24
Gastrocnemius mass	0.03	−0.04	−0.14	**0.78**	**0.78**	−**0.19**	**0.53**	**0.55**	**0.44**		−**0.48**	−0.12	−0.40	0.13
HM:BM	**0.28**	**0.32**	**0.19**	−0.50	−**0.45**	**0.21**	**0.44**	0.02	−0.10	−**0.29**		**0.50**	**0.45**	**0.45**
SM:BM	**0.20**	**0.30**	**0.20**	−0.16	−0.17	0.01	0.07	**0.83**	0.16	0.09	**0.31**		**0.49**	**0.67**
PM:BM	0.01	0.07	0.07	−0.16	−0.15	0.07	0.00	**0.19**	**0.75**	−0.11	**0.26**	**0.34**		0.27
GM:BM	**0.25**	**0.35**	**0.21**	−0.02	−0.07	−0.12	0.07	**0.36**	0.05	**0.52**	**0.21**	**0.45**	0.15	

*Numbers in bold indicate significant correlations (P < 0.05). Change, Difference between post-training and pre-training values; Gastroc, gastrocnemius; HM:BM, heart mass to body mass ratio; SM:BM, soleus mass to body mass ratio; PM:BM, plantaris mass to body mass ratio; GM:BM, gastrocnemius mass to body mass ratio. Genetic correlations were performed using strain means for each variable. Phenotypic correlations were performed using individual exercise-trained mice from all strains*.

## Discussion

The primary finding from the current study is that exercise capacity and responses to training vary considerably between the 24 inbred strains of mice tested. Baseline or intrinsic exercise capacity was 2 times higher in NOD/ShiLtJ strain compared with A/J strain. When expressed as work, mice from AKR/J strain performed significantly more work than A/J strain mice at baseline. Heritability estimates for time and work ranged from 0.85 to 0.94 and 0.82 to 0.93, respectively, suggesting that in these strains the majority of the variance in endurance exercise capacity can be attributed to genetic background. Response to training also differed between strains. The change in endurance exercise capacity ranged from −2.2 min (NON/ShiLtJ) to +8.7 min (SWR/J). The change in work also was significantly higher in mice from FVB/NJ strain compared to mice from NON/ShiLtJ strain. Based on responses from all strains, genetic background accounted for 41 to 58% of the variance in the responses to training expressed as time and from 37 to 54% when expressed as work. These results indicate genetic background significantly influences responses to exercise training. Furthermore, the data from the wide range of strains utilized in the current study are a starting point for strain selection for future studies of the genetic and physiological responses to exercise training.

Several studies have examined the influence of genetic background on intrinsic or baseline endurance exercise capacity in rodents (Barbato et al., 1998; Koch et al., 1998; Lightfoot et al., 2001; Lerman et al., 2002; Naples et al., 2010; Courtney and Massett, 2012). The most detailed work has been performed in rats selectively bred for high and low aerobic exercise capacity (Koch et al., 1998). After 20 generations of selection, rats from the high selected line run 5 times farther than those in the low selected line (Naples et al., 2010). Mice selected for voluntary wheel running also exhibit significant differences in maximal oxygen consumption and exercise capacity compared with control lines (Rezende et al., 2005, 2006; Meek et al., 2009). Endurance exercise capacity measured during a graded exercise test was approximately 20% higher in mice from selected lines vs. controls (Meek et al., 2009). In the current study, the highest performing strain ran twice as long as the lowest. This range is similar to our previous report based on 34 inbred strains (Courtney and Massett, 2012). Other studies utilizing a smaller number of strains also reported significant differences between strains. Lerman et al. reported a 75% greater maximal speed during a graded exercise test in FVB/NJ mice compared with C57BL/6J mice (Lerman et al., 2002) and Lightfoot et al. reported running duration was greater in BALB/cJ mice compared with DBA/2J (Lightfoot et al., 2001). In both studies, C57BL/6J and DBA/2J strains exhibited low exercise capacity relative to other strains. In the current study, the C57BL/6J strain was in the lower quartile for exercise time and work; however, DBA/2J strain mice were in the highest quartile of the strain distribution for both time and work. Two higher performing strains in the current study, FVB/NJ and SWR/J, were also identified in other studies as having high endurance exercise capacity (Lightfoot et al., 2001; Lerman et al., 2002). Thus, despite differences in equipment and testing protocols, a few strains have been consistently identified as having high (FVB/NJ and SWR/J) or low (C57BL/6J) endurance exercise capacity. These strains might be useful for detailed studies of the mechanisms underlying differences in endurance exercise capacity.

In contrast to the information regarding strain differences in intrinsic exercise capacity, there is less known about the influence of genetic background on the responses to exercise training in rodents. Previously, Koch et al reported a 3.9-fold difference between 10 inbred strains of rats for the change in running capacity in response to 8 weeks of treadmill running at the same absolute workload (Koch et al., 2013). In the same study, the change in distance varied from −339 m to +627 m in individual rats from a genetically heterogeneous population. Similarly, Kilikevicius et al. assessed adaptations to 5 weeks of swim training in 6 inbred strains of mice (Kilikevicius et al., 2013). Although the change in exercise capacity was not reported, post-training endurance time was approximately 6 times higher in the best performing strain compared with the lowest performing strain. In comparing swim trained and control mice within each strain, endurance exercise capacity was double in trained mice (Kilikevicius et al., 2013). Mice from three strains, C3H/HeJ, C57BL/6J, and DBA/2J, improved their endurance swimming capacity, whereas mice from A/J, BALB/cByJ, and PWD/PhJ strains showed no improvement. In our study, there were significant strain-dependent differences in the response to training expressed as change in time or work. The smallest responses to training were observed in I/LnJ, LG/J, NON/ShiLtJ, and NZW/LacJ strains. Although we previously reported the lack of response to training for NZW/LacJ mice (Massett et al., 2015), the lack of response in the other strains is a novel finding. A similar pattern was observed in a few strains of inbred rats and rats selectively bred for low responses to training (Koch et al., 2013), suggesting the negative or low responses are not training protocol or species specific. One strain in this study, A/J, showed a small response to training and had a low intrinsic exercise capacity. This strain consistently shows poor exercise capacity (Lightfoot et al., 2001; Courtney and Massett, 2012, 2014; Kilikevicius et al., 2013) and responses to training (Kilikevicius et al., 2013).

Conversely, several strains showed significant increases in endurance exercise capacity with training (129S1, CE/J, FVB/NJ, NOD/ShiLtJ, PL/J, SJL/J, and SWR/J). We, and others previously reported that FVB/NJ mice exhibit significant improvements in performance with exercise training (Massett and Berk, 2005; Chow et al., 2007; Massett et al., 2009; Gibb et al., 2016). Those changes in endurance exercise capacity were associated with physiological changes consistent with the expected cardiovascular and metabolic adaptations to training (Massett and Berk, 2005; Chow et al., 2007; Gibb et al., 2016), indicating this strain is useful for genetic and physiological studies of exercise training responses. There are few reports of training responses in the other high responding strains (Massett et al., 2015). Therefore, 129S1, CE/J, NOD/ShiLtJ, PL/J, SJL/J, and SWR/J strains should be considered for future studies of the physiological and genetic mechanisms underlying variation in endurance exercise capacity and responses to training.

To provide insight into the genetic contribution to the responses to exercise training, we calculated two estimates of broad sense heritability for endurance exercise capacity and the response to exercise training. The heritability estimates for pre- and post-training exercise time were > 0.90 and > 0.80 for *r*_*I*_ and *g*^2^, respectively (Table 1), suggesting that a high proportion of the total variance in endurance exercise capacity is due to genetic background. These estimates are higher than our prior estimates based on a smaller number of mouse strains (Massett and Berk, 2005). Broad sense heritability estimates for the change in time (*r*_*I*_ = 0.58 and *g*^2^ = 0.41) were smaller than for pre- and post-training exercise capacity. Estimates for the current study are similar to those previously reported for changes in time (0.55 and 0.38) and work (0.55 and 0.38) in inbred and hybrid mouse strains (Massett and Berk, 2005). They also are consistent with the heritability estimates for changes in VO_2max_ with exercise training in humans (Prud'homme et al., 1984; Hamel et al., 1986; Bouchard et al., 1999; Zadro et al., 2017). The relatively high heritability and large strain differences in the responses to training observed in the current study suggest this phenotype is influenced by genetic factors and these mouse strains would be amenable to more detailed genetic (i.e., linkage analysis or genome-wide association studies) and physiological studies of the mechanisms underlying variation in responses to exercise training.

In addition to genetic background, there are several possible factors influencing the response to training. In humans and rodents, the magnitude of the training response can vary with exercise intensity or study duration (Kemi et al., 2002; Koch et al., 2005; Sisson et al., 2009; Gibb et al., 2016; Montero and Lundby, 2017). Additional studies are required to determine if varying the training paradigm improves responses in low responding strains from this study. However, some rodent strains might be resistant to moderate exercise training. For example, Koch et al. (2005) reported that COP rats showed little or no improvement in exercise capacity in response to two different exercise training intensities, suggesting this strain is not responsive to moderate treadmill running. That study also addressed the principle of initial values, reporting that pre-training exercise capacity did not influence training responses in two inbred rat strains (Koch et al., 2005). In contrast, in our current study, baseline exercise capacity was significantly related to the change in exercise capacity and the regression coefficient for baseline exercise capacity indicated a negative association between baseline exercise capacity and the response to training. Thus, baseline endurance exercise capacity might play a role in the response to training in the mouse strains tested. The rate of growth and development over the course of the study might have influenced the responses to training as well. Although the change in body mass was not significantly correlated with the change in time, mice from both groups gained 3–4 g over the course of the study and there were significant strain differences in the change in body mass (Figure 3B). The increase in body mass suggests that the mice continued to grow and develop over the course of the study, which could impact the response to training. For example, the hypertrophic response to synergist ablation overload is different between young (10 week-old) and mature (>16 week-old) mice, indicating that adaptation to exercise is influenced by maturational age (Murach et al., 2017). Therefore, the lack of observed differences in skeletal muscle mass between EX and SED groups could be related to the relatively young age of the mice used in the current study. Additional studies are required to determine if the adaptations to endurance exercise training are both strain- and age-dependent.

An important aspect of the current study is the inclusion of SED mice for each strain. SED mice were included as time controls and to contrast with responses in EX mice of the same strain. As a whole, SED mice exhibited a small decrease in exercise time (−0.79 ± 3.25 min) over the 4-week training period. The small average change in endurance exercise capacity is similar to our previous data from fewer inbred and hybrid mouse strains (Massett and Berk, 2005). Although larger changes are atypical for SED mice, significant decreases in exercise capacity with time have been reported for inbred rats and mice over an 8-week training period (Koch et al., 2005; Ferreira et al., 2007). Knowing the variation in the SED group also provided an estimate of the typical error (TE). Changes in endurance exercise capacity beyond two times TE suggest there is a high probability that this is a true physiological change (Hopkins, 2000). Using twice the TE, we identified several EX strains as having positive responses to training, based on the change in time and the change in work. Thus, the results of this study reinforce the importance of including SED or sham controls in exercise training studies to interpret training adaptations and assist in selection of high and low responding strains for genetic and physiological studies.

In summary, this study demonstrates there are considerable differences between the 24 inbred mouse strains tested in exercise capacity and the change in endurance exercise capacity. These differences between inbred strains combined with the high broad sense heritability estimates support the contribution of a genetic component to the variation in exercise capacity and responses to training in mice. These results provide a foundation for mouse strain selection to investigate the physiological adaptations to exercise as well as genetic modifiers of exercise capacity and responses to exercise training.

## Author contributions

Conceived and designed the experiments: JA, SK, and MM. Performed the experiments: JA and SK. Analyzed the data: JA and MM. Drafted, edited, and revised manuscript: JA, SK, and MM. Approved final version of manuscript: JA, SK, and MM.

### Conflict of interest statement

The authors declare that the research was conducted in the absence of any commercial or financial relationships that could be construed as a potential conflict of interest.
