# Supplementary material for: Differences in Exercise Capacity and Responses to Training in 24 Inbred Mouse Strains
Source: Front Physiol. 2017 Nov 30;8:974. doi: 10.3389/fphys.2017.00974 (PMC5714923; doi:10.3389/fphys.2017.00974)
Supplement: Supplementary file 1 [file Table1.docx]

**Supplemental Table 1.** Least squares means for strain by group interaction from ANCOVA to determine strain and group differences controlling for baseline exercise time.

|  |  |  |  | 99% CI | |
| --- | --- | --- | --- | --- | --- |
| Strain | Group | LSM | SE | Lower | Upper |
| 129S1/SvlmJ | EX | 0.06 | 0.01 | 0.03 | 0.09 * † |
|  | SED | -0.04 | 0.01 | -0.08 | -0.01 * |
| 129X1/SVJ | EX | -0.01 | 0.01 | -0.04 | 0.03 |
|  | SED | 0.02 | 0.01 | -0.02 | 0.05 |
| A/J | EX | -0.08 | 0.02 | -0.14 | -0.02 |
|  | SED | -0.12 | 0.02 | -0.18 | -0.06 * |
| AKR/J | EX | 0.10 | 0.01 | 0.06 | 0.14 * |
|  | SED | 0.03 | 0.01 | -0.01 | 0.07 |
| BALB/cByJ | EX | 0.05 | 0.01 | 0.01 | 0.08 * |
|  | SED | 0.00 | 0.01 | -0.03 | 0.04 |
| C3H/HeJ | EX | 0.03 | 0.01 | -0.01 | 0.06 |
|  | SED | -0.04 | 0.01 | -0.08 | 0.00 |
| C57BL/6J | EX | 0.00 | 0.01 | -0.04 | 0.04 |
|  | SED | -0.06 | 0.01 | -0.11 | -0.02 |
| C57BR/cdJ | EX | 0.05 | 0.01 | 0.01 | 0.09 * |
|  | SED | 0.01 | 0.01 | -0.03 | 0.04 |
| CBA/J | EX | -0.01 | 0.01 | -0.05 | 0.03 † |
|  | SED | -0.09 | 0.01 | -0.13 | -0.04 * |
| CE/J | EX | 0.07 | 0.01 | 0.03 | 0.10 * † |
|  | SED | -0.06 | 0.01 | -0.09 | -0.02 * |
| DBA/2J | EX | 0.07 | 0.01 | 0.03 | 0.11 * |
|  | SED | 0.06 | 0.01 | 0.02 | 0.09 * |
| FVB/NJ | EX | 0.11 | 0.01 | 0.08 | 0.14 * † |
|  | SED | 0.03 | 0.01 | 0.00 | 0.06 |
| I/LnJ | EX | -0.04 | 0.01 | -0.08 | 0.00 |
|  | SED | -0.08 | 0.01 | -0.12 | -0.04 * |
| LG/J | EX | -0.04 | 0.01 | -0.09 | 0.00 |
|  | SED | -0.02 | 0.01 | -0.06 | 0.03 |
| LP/J | EX | 0.02 | 0.01 | -0.02 | 0.05 |
|  | SED | 0.01 | 0.01 | -0.03 | 0.04 |
| MA/MyJ | EX | 0.02 | 0.01 | -0.02 | 0.05 |
|  | SED | 0.03 | 0.01 | -0.01 | 0.06 |
| NOD/ShiLtJ | EX | 0.10 | 0.01 | 0.05 | 0.14 * † |
|  | SED | -0.01 | 0.01 | -0.05 | 0.03 |
| NON/ShiLtJ | EX | -0.02 | 0.01 | -0.05 | 0.02 |
|  | SED | -0.05 | 0.01 | -0.09 | -0.02 * |
| NZW/LacJ | EX | -0.09 | 0.01 | -0.13 | -0.04 * |
|  | SED | -0.07 | 0.01 | -0.11 | -0.02 * |
| PL/J | EX | 0.06 | 0.01 | 0.03 | 0.10 * † |
|  | SED | -0.02 | 0.01 | -0.06 | 0.02 |
| PWD/PhJ | EX | 0.12 | 0.01 | 0.08 | 0.16 * |
|  | SED | 0.09 | 0.01 | 0.05 | 0.14 * |
| SJL/J | EX | 0.13 | 0.01 | 0.09 | 0.17 * † |
|  | SED | 0.03 | 0.01 | -0.01 | 0.06 |
| SM/J | EX | 0.07 | 0.01 | 0.03 | 0.11 * |
|  | SED | 0.02 | 0.01 | -0.02 | 0.06 |
| SWR/J | EX | 0.12 | 0.01 | 0.08 | 0.16 * † |
|  | SED | 0.01 | 0.01 | -0.04 | 0.05 |

ANCOVA was performed using log-transformed data for baseline (pre) exercise time and the change in time. EX, exercise-trained; SED, sedentary control; LSM, Least Squares Mean; SE, Standard Error, Lower, Lower bound of 99.9% confidence interval (CI); Upper, Upper bound of 99.9% confidence interval (CI). *, Significant increase or decrease in exercise capacity; †, Significant difference between EX and SED, P < 0.001.
