# Supplementary material for: Differences in Exercise Capacity and Responses to Training in 24 Inbred Mouse Strains
Source: Front Physiol. 2017 Nov 30;8:974. doi: 10.3389/fphys.2017.00974 (PMC5714923; doi:10.3389/fphys.2017.00974)
Supplement: Supplementary file 2 [file Table2.docx]

**Supplemental Table 2.** Least squares means for strain by group interaction from ANCOVA to determine strain and group differences controlling for baseline work.

|  |  |  |  | 99% CI | |
| --- | --- | --- | --- | --- | --- |
| Strain | Group | LSM | SE | Lower | Upper |
| 129S1/SvlmJ | EX | 0.21 | 0.03 | 0.11 | 0.32 * † |
|  | SED | -0.08 | 0.03 | -0.19 | 0.02 |
| 129X1/SVJ | EX | 0.02 | 0.03 | -0.08 | 0.12 |
|  | SED | 0.12 | 0.03 | 0.02 | 0.22 * |
| A/J | EX | -0.20 | 0.05 | -0.38 | -0.02 * |
|  | SED | -0.35 | 0.05 | -0.53 | -0.17 * |
| AKR/J | EX | 0.34 | 0.04 | 0.21 | 0.47 * |
|  | SED | 0.18 | 0.04 | 0.06 | 0.30 * |
| BALB/cByJ | EX | 0.22 | 0.03 | 0.11 | 0.32 * |
|  | SED | 0.09 | 0.03 | -0.01 | 0.20 |
| C3H/HeJ | EX | 0.14 | 0.03 | 0.04 | 0.24 * † |
|  | SED | -0.10 | 0.03 | -0.22 | 0.02 |
| C57BL/6J | EX | 0.09 | 0.04 | -0.03 | 0.21 |
|  | SED | -0.10 | 0.04 | -0.22 | 0.02 |
| C57BR/cdJ | EX | 0.20 | 0.04 | 0.08 | 0.32 * |
|  | SED | 0.14 | 0.04 | 0.02 | 0.26 * |
| CBA/J | EX | 0.05 | 0.03 | -0.06 | 0.17 |
|  | SED | -0.12 | 0.04 | -0.23 | 0.00 |
| CE/J | EX | 0.25 | 0.03 | 0.14 | 0.35 * † |
|  | SED | -0.06 | 0.03 | -0.17 | 0.04 |
| DBA/2J | EX | 0.23 | 0.04 | 0.11 | 0.35 * |
|  | SED | 0.23 | 0.03 | 0.12 | 0.33 * |
| FVB/NJ | EX | 0.37 | 0.03 | 0.28 | 0.46 * † |
|  | SED | 0.17 | 0.03 | 0.07 | 0.27 * |
| I/LnJ | EX | -0.07 | 0.03 | -0.18 | 0.05 |
|  | SED | -0.21 | 0.04 | -0.33 | -0.09 * |
| LG/J | EX | 0.06 | 0.04 | -0.07 | 0.19 |
|  | SED | 0.17 | 0.04 | 0.04 | 0.29 * |
| LP/J | EX | 0.10 | 0.03 | -0.01 | 0.20 |
|  | SED | 0.09 | 0.03 | -0.02 | 0.19 |
| MA/MyJ | EX | 0.09 | 0.03 | -0.02 | 0.19 |
|  | SED | 0.14 | 0.03 | 0.04 | 0.25 * |
| NOD/ShiLtJ | EX | 0.31 | 0.04 | 0.19 | 0.43 * † |
|  | SED | 0.04 | 0.04 | -0.08 | 0.17 |
| NON/ShiLtJ | EX | 0.09 | 0.03 | -0.02 | 0.20 |
|  | SED | -0.04 | 0.03 | -0.14 | 0.07 |
| NZW/LacJ | EX | -0.18 | 0.04 | -0.30 | -0.06 * |
|  | SED | -0.10 | 0.04 | -0.22 | 0.02 |
| PL/J | EX | 0.24 | 0.03 | 0.14 | 0.35 * † |
|  | SED | -0.05 | 0.03 | -0.16 | 0.07 |
| PWD/PhJ | EX | 0.23 | 0.03 | 0.13 | 0.33 * |
|  | SED | 0.14 | 0.03 | 0.03 | 0.26 * |
| SJL/J | EX | 0.34 | 0.03 | 0.23 | 0.45 * |
|  | SED | 0.15 | 0.03 | 0.04 | 0.25 * |
| SM/J | EX | 0.24 | 0.03 | 0.14 | 0.35 * |
|  | SED | 0.11 | 0.03 | 0.00 | 0.22 |
| SWR/J | EX | 0.36 | 0.03 | 0.24 | 0.47 * † |
|  | SED | 0.09 | 0.04 | -0.03 | 0.21 |

ANCOVA was performed using log-transformed data for baseline (pre) work and the change in work. EX, exercise-trained; SED, sedentary control; LSM, Least Squares Mean; SE, Standard Error, Lower, Lower bound of 99.9% confidence interval (CI); Upper, Upper bound of 99.9% confidence interval (CI). *, Significant increase or decrease in exercise capacity; †, Significant difference between EX and SED, P < 0.001.
